# Quantitative Analysis of MicroRNAs in *Vaccinia virus* Infection Reveals Diversity in Their Susceptibility to Modification and Suppression

**DOI:** 10.1371/journal.pone.0131787

**Published:** 2015-07-10

**Authors:** Amy H. Buck, Alasdair Ivens, Katrina Gordon, Nicola Craig, Alexandre Houzelle, Alice Roche, Neil Turnbull, Philippa M. Beard

**Affiliations:** 1 Centre for Immunity, Infection and Evolution, University of Edinburgh, King’s Buildings, Edinburgh, United Kingdom; 2 Infection and Immunity, The Roslin Institute / Royal (Dick) School of Veterinary Studies, Easter Bush, Midlothian, United Kingdom; University of Liverpool, UNITED KINGDOM

## Abstract

*Vaccinia virus* (VACV) is a large cytoplasmic DNA virus that causes dramatic alterations to many cellular pathways including microRNA biogenesis. The virus encodes a poly(A) polymerase which was previously shown to add poly(A) tails to the 3’ end of cellular miRNAs, resulting in their degradation by 24 hours post infection (hpi). Here we used small RNA sequencing to quantify the impact of VACV infection on cellular miRNAs in human cells at both early (6 h) and late (24 h) times post infection. A detailed quantitative analysis of individual miRNAs revealed marked diversity in the extent of their modification and relative change in abundance during infection. Some miRNAs became highly modified (e.g. miR-29a-3p, miR-27b-3p) whereas others appeared resistant (e.g. miR-16-5p). Furthermore, miRNAs that were highly tailed at 6 hpi were not necessarily among the most reduced at 24 hpi. These results suggest that intrinsic features of human cellular miRNAs cause them to be differentially polyadenylated and altered in abundance during VACV infection. We also demonstrate that intermediate and late VACV gene expression are required for optimal repression of some miRNAs including miR-27-3p. Overall this work reveals complex and varied consequences of VACV infection on host miRNAs and identifies miRNAs which are largely resistant to VACV-induced polyadenylation and are therefore present at functional levels during the initial stages of infection and replication.

## Introduction

MicroRNAs have emerged as important regulators of protein expression, influencing many biological pathways including those associated with viral infection [1]. These small RNAs function by guiding the RNA-induced silencing complex (RISC) to messenger RNAs (mRNAs) with base pair complementarity, resulting in inhibition of translation and/or mRNA destabilization [2]. The biogenesis of a miRNA (reviewed in [3, 4]) begins in the nucleus with synthesis of a primary miRNA transcript (pri-miRNA), generally by RNA polymerase II. In mammals the Drosha nuclear RNase III endonuclease and DGCR8 process the pri-miRNA to a shorter (approximately 70 nt) hairpin structure known as the precursor miRNA (pre-miRNA). This structure is transported to the cytoplasm via exportin 5, where it is processed to a ~ 22 nt duplex RNA by the RNase Dicer and its cofactor TRBP. The RNA duplex is loaded into the Argonaute (Ago) proteins, where one strand (the passenger or star strand) is released and degraded, and the other strand (the guide) retained. The guide strand then targets mRNAs primarily through complementarity at positions 2–8 of the 5′ end of the miRNA (termed the “seed”) [5].

Several non-canonical miRNA biogenesis pathways have been described that include the ability to bypass the need for processing in the nucleus [6]. Some viruses have evolved to express their own miRNAs by these canonical or non-canonical pathways [7] and viruses can also be engineered to produce miRNAs [8, 9]. Poxviruses are large cytoplasmic DNA viruses with a complex life cycle that includes viral DNA replication and transcription occurring in specialised cytoplasmic “viral factories” by virally-encoded polymerase enzymes [10]. VACV is the prototypic orthopoxvirus which does not encode any viral miRNAs [1] but induces polyadenylation of mature cellular miRNAs with a concurrent widespread reduction in abundance by 24 h pi [11, 12].

Dysregulation of miRNA expression has been linked with numerous diseases [13] and there is extensive interest in understanding how the levels of these molecules are naturally regulated. While the steps leading to miRNA production and maturation are relatively well understood [14] the mechanisms involved in the decay of miRNAs remain somewhat elusive [15, 16]. In many cellular contexts mature miRNAs appear to be extremely stable with half-lives in the range of days [17–19]. However some miRNAs exhibit rapid downregulation under certain physiological conditions including cell cycle, neuronal activation, viral infection or in response to growth factors [15]. This implies regulated mechanisms for miRNA decay. Several studies in animals have suggested some exonucleases associated with degradation of the miRNAs, reviewed in [15, 20]. Loss of Ago2 in mammals also results in a reduction in miRNA levels, suggesting Ago2 provides a level of protection and stabilisation of the mature miRNAs [21, 22].

One mechanism for selectively degrading a specific miRNA involves recognition of the mature sequence by another non-coding RNA; this has been termed “target mediated miRNA degradation” (TMMD) and requires extensive complementarity between the two RNAs[23]. The mechanisms associated with TMMD are still emerging. For example in *Drosophila* and human cell lines miRNA destabilisation is accompanied by the emergence of longer “tailed” or shorter “trimmed” versions of the miRNA but relatively little is known about how the two events are linked [23–25]. Sequencing analyses in a variety of systems have revealed the ubiquity of miRNA isomers that vary in length and the extent of non-templated additions (NTAs) at their 3’ end [26, 27]. The functional effect of these modifications appears to be context dependent with examples of both stabilization and increased degradation as a result of modifications [15, 28]. In mammalian cells, for example, a single additional adenosine at the 3’ end of miR-122 can have a stabilizing effect [29] whereas addition of uridines to miR-26 can accelerate its decay [30].

Here we aimed to analyse the changes occurring to endogenously expressed miRNAs (<40 nucleotides) in the face of VACV infection at both early (6 h) and late (24 h) times post infection. We found the extent of VACV-induced modification of cellular miRNAs was diverse, as was the extent of reduction in their steady state levels, particularly at 6 hpi. Adenylation and polyadenylation of the 3’ end of miRNAs was common but varied substantially across individual miRNAs and did not correlate with the observed reduction in abundance. Interruption of the viral life cycle resulted in accumulation of some cellular miRNAs, indicating the possibility of regulation of miRNA levels by intermediate and/or late VACV gene expression.

## Materials and Methods

### Cells, virus and antibodies

Human cervix carcinoma epithelial cells (HeLa) and Chinese Hamster Ovary K1 (CHO) cells were grown in Dulbecco’s modified Eagle’s medium (DMEM) (Life Technologies) containing 50 IU/ml penicillin, 50 μg/ml streptomycin (Sigma) and 10% foetal bovine serum (FBS) (Life Technologies). Cells were incubated at 37°C in a 5% CO_2_ incubator. The experiments presented here were carried out with sucrose gradient-purified IMV form of VACV strain Western Reserve (WR) or a genetically modified Modified Vaccinia virus Ankara strain expressing a protozoan *Theileria parva* TP2 antigen (MVA).

### Preparation of RNA samples for sequencing analysis

Confluent 175cm^2^ flasks of HeLa cells were infected with VACV at an MOI of 10 for 1 h at 37°C. The inoculum was removed (time point 0 h), cells were washed and incubated with DMEM with 2.5% FBS. At 6 and 24 hpi cells were washed once with ice cold PBS then collected into an appropriate volume of QIAzol lysis reagent (Qiagen) and frozen at -80°C. Cytosine D-arabinofuranoside (AraC) was added to a final concentration of 40μg/ml at time point 0 h to a parallel set of samples. RNA was extracted using the miRNAeasy kit (Qiagen), the integrity of the RNA measured as ≥ 9.5 for all samples based on the Bioanalyzer (Agilent) and 10 μg of RNA was gel purified (20–40 nt) by 15% UREA PAGE prior to library preparation with the TrueSeq kit (Illumina).

### Sequencing analysis

Libraries were pooled and sequenced on the Miseq Illumina platform yielding an average of 632,604 ± 94,061 reads per sample. Total reads were assessed for quality using fastQC version 0.10.1 and processed using cutadapt v1.1 (parameters:-O 9-m 16-n 4-q 20). Fasta sequences of 16 or more nts were extracted from the primer-trimmed reads and collapsed to generate a non-redundant set of sequences within each sample. A minimum threshold of 2 identical reads within a given sample was applied for collapsing. Collapsed sequences were assessed for human genome (hg19) matches using the mirDeep2 mapper.pl script [31], filtered to require a perfect match along the entire read length (full length perfect match (FLPM)). Those reads that perfectly aligned were then assessed for ncRNA content by similarity searches (using BLASTN) against the Rfam database allowing up to 2 mismatches. Unclassified sequences and “miRNA” classified sequences from the Rfam scan were subsequently analysed for miRNA content using bowtie (v0.12.5, parameters:-p 20-f-n 0-e 80-l 50-a-m 500—norc—chunkmbs 256—best—strata) with a list of mature miRNA sequences from mirBase v19, extended to the 26 nt templated sequence “26-base mature”. Those exhibiting FLPM were retained. Sequencing data was deposited in GEO database (GSE54235).

### Differential expression analysis and identification of modifications

Analysis of miRNA differential expression was carried out in R/Bioconductor using the bowtie alignments as input. Only miRNAs identified to be present at an abundance level of greater than 0.01% (100 reads per million) in at least one of the samples were analysed. Differential expression was assessed using the limma Bioconductor package, after conversion of abundances to log2-based tag counts. Pair-wise comparisons of sample groups were performed as appropriate, with p value adjustment for multiple testing. In two cases the input sequences exhibited perfect alignment to 2 or more full length (22 nt) mature miRNAs: let-7f-5p and miR-196a-5p; these were excluded from further analysis to avoid complication in mapping total reads to each miRNA, given the presence of both short (ambiguous) and long (locus-specific) isoforms.

The reads that did not align perfectly over their full length to the human genome were considered candidates for 3’ end modification. These were first subsetted by the requirement that the first 19 nt had to align perfectly to the first 19 nt of the mature sequence (with no restriction on subsequent mismatches). Sequences meeting this criterion, i.e. they aligned to at least the first 19 nt of a known miRNA but contained at least one mismatch after this position were deemed "modified reads" and were then further classified based on the identity of their modifications and incidence of sequential A residues.

### Northern blot analysis

Northern blot analysis was carried out as described in[32]. Briefly, 7.5 ug of total RNA was loaded onto 15% polyacrylamide gels, run in 0.5% TBE for 2 hours, after which total RNA levels were visualized by staining with ethidium bromide to check for equal loading. An example of an image of an ethidium bromide-stained gel is provided in S5 Fig. The gels were then transferred to Hybond N (Amersham) membrane for 1 hr at 4°C, 80 V and crosslinking carried out as described in [33]. Membranes were pre-hybridized in PerfectHyb (Sigma) prior to incubation with P^32^-labeled probes. The DNA probes were perfectly complementary to the mature miRNA sequence and were labelled with T4 PNK (Invitrogen) following manufacturer's instructions.

## Results

### VACV infection causes widespread reduction of cellular miRNAs at 24 hpi but has varied effects at 6 hpi

VACV has previously been shown to degrade both exogenous and endogenous miRNAs in human cells via addition of a 3’ polyadenosine (polyA) tail catalysed by the VACV polymerase VP55 [11]. This previous study analysed miRNA abundance at a late time point post infection (24 h) when most cellular processes are dramatically altered, the cell contains abundant mature and immature virions and is near death. We therefore sought to extend this work to an earlier time point during infection (6 h) to understand whether miRNAs can indeed be rapidly modified and reduced during the initial stages of viral replication. Samples were collected at both early (6 h) and late (24 h) time points post infection as well as in the presence or absence of Cytosine D-arabinofuranoside (AraC), a drug that inhibits the VACV replication cascade after early viral gene expression by blocking DNA replication and thus dramatically reducing the amount of template for intermediate and late RNA expression. This comparison enables us to examine whether intermediate and late viral gene products influence the extent to which miRNAs are suppressed.

**Fig 1:**
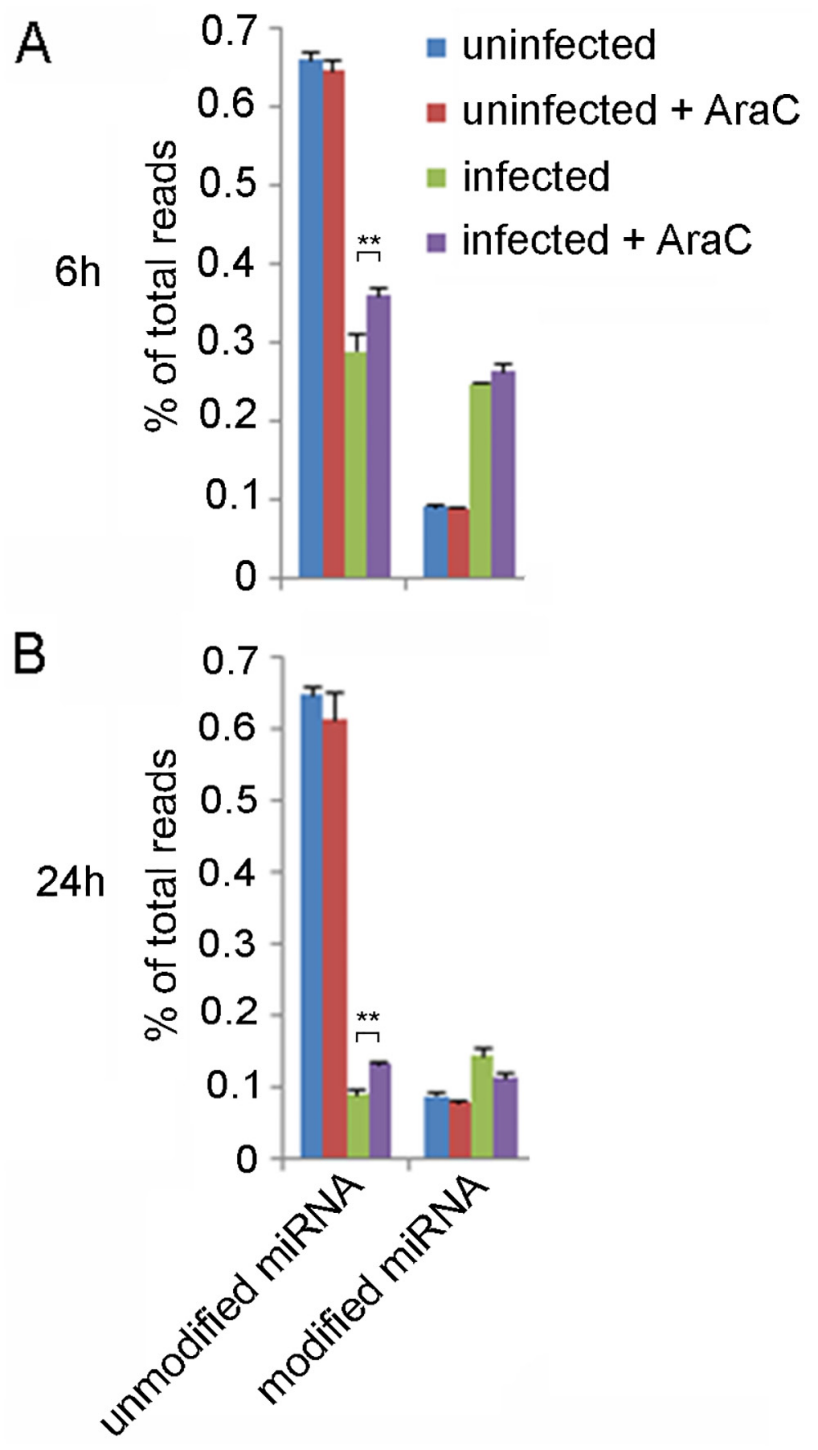
VACV causes a marked reduction in unmodified miRNA reads and an increase in modified miRNA reads in HeLa cells at (A) 6 and (B) 24 hpi. The miRNA content of HeLa cells infected with VACV or mock infected, with and without addition of AraC, at 6 and 24 hpi. The proportion of reads is displayed as the percentage of total read sequences (that passed quality control criteria) in each condition (average of three replicates). Modified reads are defined as those containing a non-templated nucleotide after position 19 in the mature sequence. Error bars represent SD (n = 3 biological replicates). The increase in unmodified miRNAs in infected cells in response to AraC treatment at 6 and 24 hpi is statistically significant as shown (Student’s t-Test, p value <0.01). The data graphed in this figure are detailed in S2 Table.

Small RNA populations from three biological replicates were sequenced for each of the following treatments: (i) mock infection, (ii) mock infection in the presence of AraC, (iii) infection with VACV or (iv) infection with VACV in the presence of AraC. The majority of reads in each sample perfectly matched the human genome (57–82%; S1 Fig and S1 Table) and aligned to human miRNAs. These were classified into two categories: “unmodified”, which perfectly matched the genomic sequence (accounting for length heterogeneity but excluding non-templated additions to the 3’ end) and “modified”, which contained at least 1 non-templated nucleotide (nt) at the 3’ end of the mature sequence (defined here as after nt position 19). At both 6 and 24 hpi there was a marked decrease in the abundance of unmodified miRNAs in VACV-infected compared to uninfected cells, with a 2.3 fold reduction at 6 hpi and 7.3 fold reduction at 24 hpi (Fig 1). The reduction was significantly less in cells treated with AraC, with 1.8 and 5.0 fold decreases in unmodified miRNAs at 6 and 24 hpi, respectively. Similar to previous observations in human cells [24, 26, 27] we found that a fraction of miRNA reads (12%) contained non-templated nts at their 3’ ends in uninfected cells (Fig 1 and S2 Table). Consistent with the report by Backes et al., 2012, this proportion increased in response to VACV infection (Fig 1).

**Fig 2:**
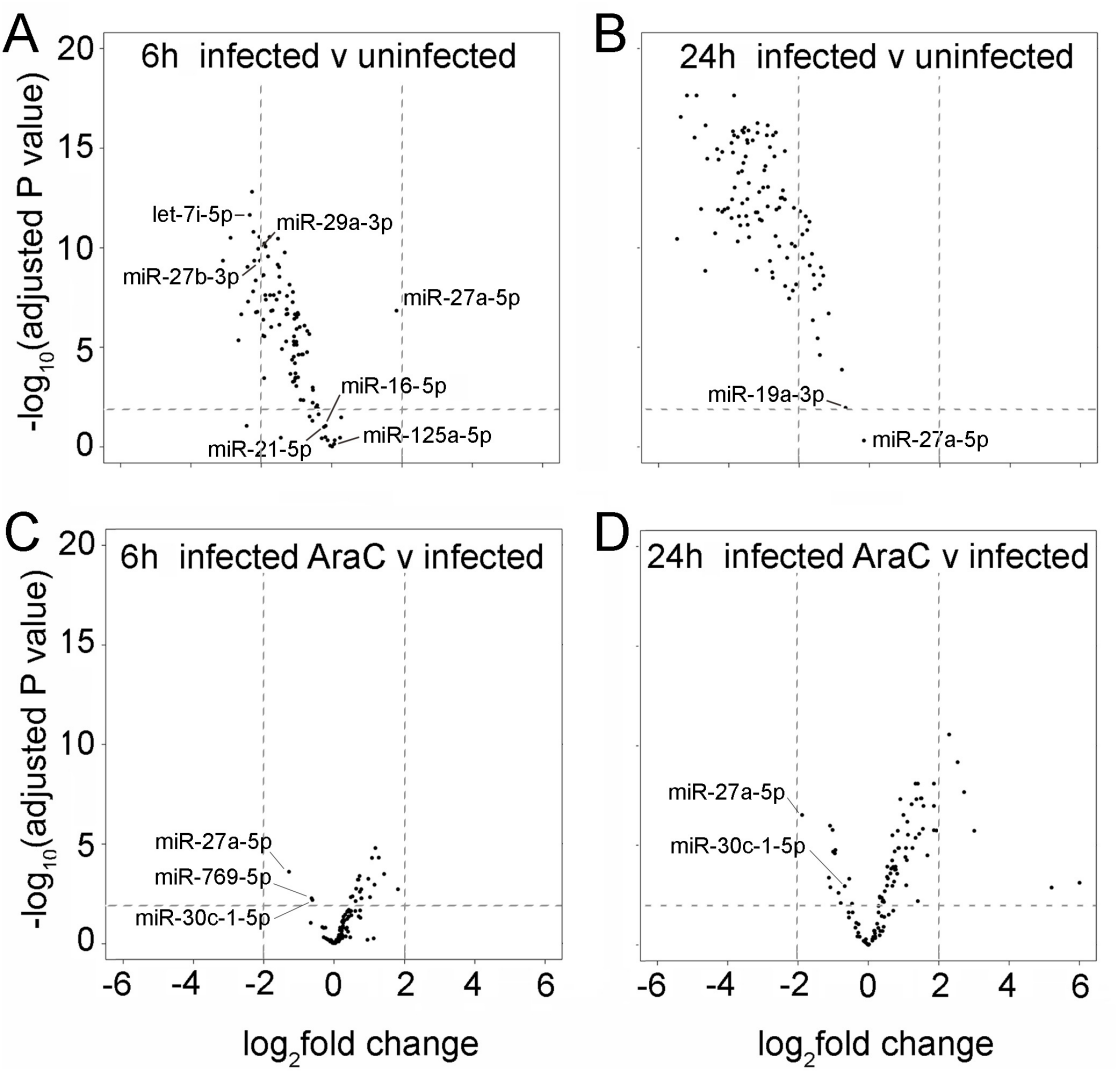
VACV causes variable effects on the levels of unmodified miRNAs at 6 and 24 hpi. Four Volcano plots show the differential expression levels of the 107 most highly expressed cellular miRNAs (“highly expressed” is based on a cut-off of >100 reads per million reads that aligned to the human genome). The x axes represent the fold change of the miRNA and the y axis the FDR p value associated with the fold change. The plots portray the alteration in abundance of unmodified miRNAs in VACV-infected cells compared to uninfected cells at (A) 6 hpi and (B) 24 hpi, and in VACV-infected cells with AraC compared to VACV-infected cells without AraC at (C) 6 hpi and (D) 24 hpi. The horizontal dotted line delineates a FDR p value of 0.01. The vertical lines delineate a fold change of ±log_2_2. The miRNAs that are discussed in the manuscript are indicated.

Using the replicate data we further analyzed changes in individual miRNAs present at sufficient abundance levels to enable statistical analysis. The miRNAs analysed here for differential expression were present at a relative level of > 100 reads per million reads sequenced (counting only those that aligned to the human genome). Out of the 526 human miRNAs in HeLa cells that were detected, 107 miRNAs fit these criteria. These 107 miRNAs were analysed for differential expression and the results displayed on a volcano plot (Fig 2) which shows the fold change in abundance of the unmodified miRNA (x axis) compared to the p value associated with this change (y axis). As shown in Fig 2, at 6 hpi VACV infection caused a significant reduction (False Discovery Rate p < 0.01) in 82% of these miRNAs, with 67% reduced at least 2 fold. This was not a global phenomenon, however, as the levels of 17% of the miRNAs were not significantly changed at 6 hpi (FDR p>0.01 in Fig 2a; S3 Table), revealing that individual miRNAs exhibit differing responses to VACV infection. One miRNA, miR-27a-5p, was significantly up-regulated in response to VACV infection. Further examples of miRNAs which increased at 6 hpi within the entire dataset of 526 miRNAs included miR-92a-5p, miR-501-3p, miR-132-3p, miR-132-5p, and miR-203-5p (S2 Fig and S4 Table). Less variation in the response to VACV infection was observed at 24 hpi, when 97% of the miRNAs were significantly reduced (Fig 2b) however miR-27a-5p remains present at levels comparable to uninfected cells even at this time point. This miRNA was previously annotated as a passenger strand and, although in the top 107 most abundant miRNAs, is relatively lowly expressed (135 reads per million in uninfected cells). The annotation of a “passenger strand” is complex since recent reports have shown that the proportions of guide and passenger strands are cell and tissue-specific and many passenger strands are conserved and functional [34]. Here we use “5p and 3p” nomenclature to avoid confusion. Backes et al. found that the passenger strand of exogenously over-expressed miR-124 was not polyadenylated in VACV-infected cells [11], however in our dataset we find examples of modification and polyadenylation of both 5p and 3p strands of some miRNAs, including some which have been annotated as “mature” and “passenger” based on the historical definition (where there is >6 fold difference in the abundance level of 5p and 3p strands), for example miR-21-3p and miR-30a-3p (S5 and S6 Tables). As another example, we see comparable levels of infection-induced tailing on 5p and 3p arms of miR-28 (S5 Table), even though the 3p arm is present at a > 8 fold higher abundance compared to the 5p arm (S6 Table). Overall there is no obvious correlation between abundance level in the uninfected cells and extent of modification at 6 hpi (data not shown).

### Limiting VACV replication with AraC significantly alters the extent of virus-induced miRNA reduction

In order to probe the potential contributions of viral factors in the reduction of cellular miRNAs induced by VACV we compared miRNA levels in cells infected with VACV in the absence or presence of the DNA replication inhibitor AraC. VACV gene expression is temporally regulated and can be divided into early, intermediate and late phases. AraC arrests the replication cycle of the virus at the end of the early phase, dramatically reducing intermediate and late VACV gene expression [35]. As shown in Fig 2c, in cells treated with AraC there was a significant increase in 20 out of the 107 (19%) most highly expressed miRNAs at 6 hpi. This effect was more prominent at 24 hpi, with 47 miRNAs (44%) at significantly higher levels in the presence of AraC (Fig 2d), indicating a complete VACV replication cycle is required to achieve optimal reductions in miRNAs. Treatment of uninfected cells with AraC did not affect the level of any miRNA, ruling out non-specific effects of drug treatment (S3 Fig). Furthermore, the abundance of 3 miRNAs (miR-27a-5p, miR-769-5p and miR-30c-1-5p) significantly decreased in response to AraC treatment of infected cells at 6 hpi (Fig 2c). Thus AraC does not globally inhibit VACV-induced miRNA reduction but instead has specific effects on individual miRNAs in infected cells. Overall, these results suggest diversity in the susceptibility of miRNAs to alterations in abundance associated with VACV infection and the temporal mechanisms involved in this regulation.

### *Vaccinia virus* early gene expression results in 3’ polyadenylation of the majority of host miRNAs

**Fig 3:**
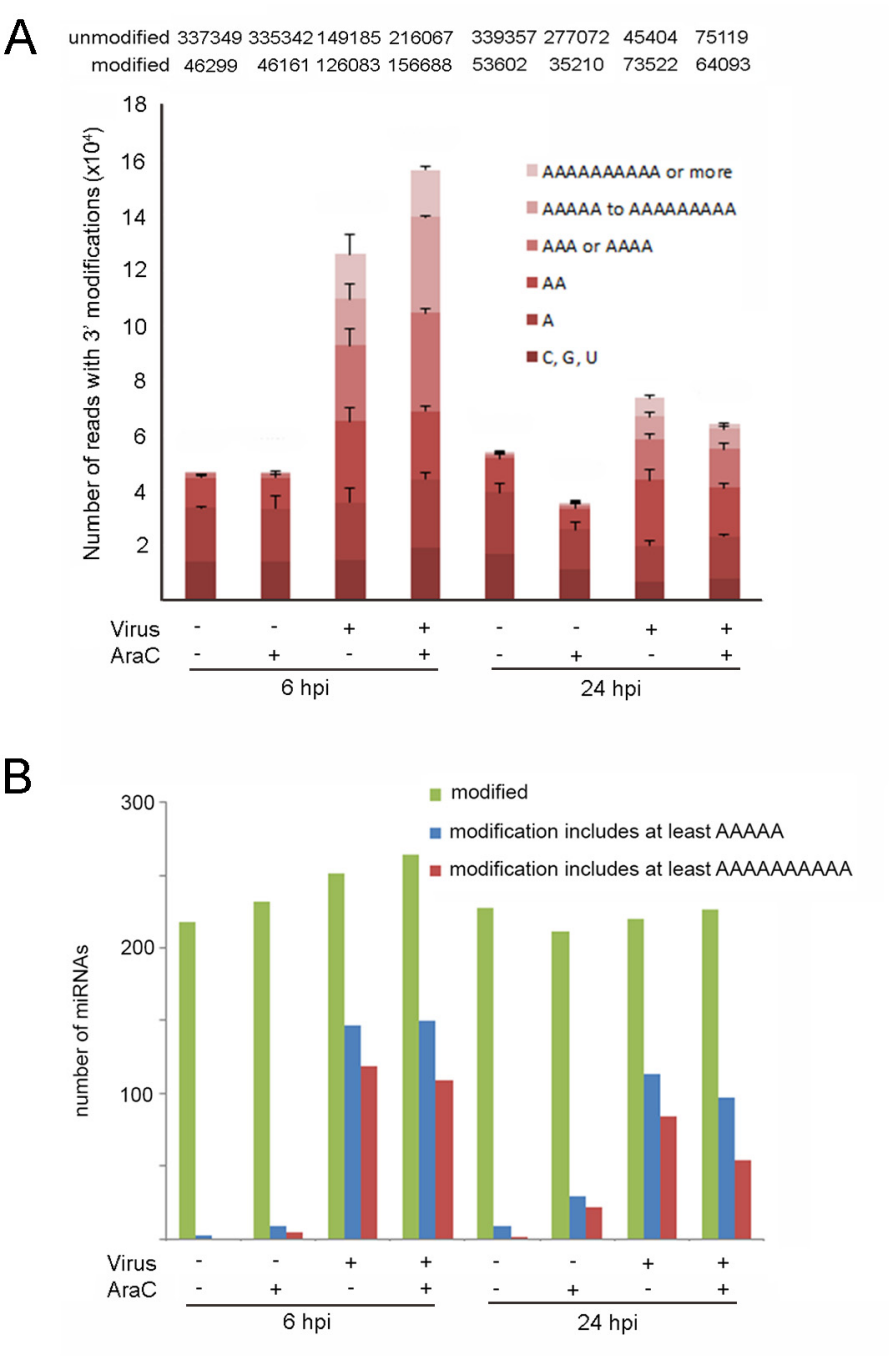
VACV generates long 3’ polyA extensions to endogenous miRNAs. (A) The 3’ modifications of miRNAs (defined by a non-templated nucleotide after position 19 in the mature sequence) were classified by the nucleotide identity (C, G or U) or A, which was then further classified by the number of sequential adenosines: 1, 2, 3–4, 5–9 or 10+. The number of reads plotted on the Y axis are the average of 3 replicates and error bars indicate standard deviation. The actual number of reads in each condition is noted above the column. (B) The number of distinct miRNAs with at least 2 modified reads detected is indicated in green; of these, the miRNAs showing modifications with at least 5 sequential adenosines are depicted in blue or modified by at least 10 sequential adenosines in red.

In parallel with the reduction of unmodified miRNA reads we detected a marked increase in modified miRNA reads in VACV infected cells (Fig 1). Modified reads were defined as containing a non-templated nt at position 19 or higher in the mature sequence (Materials and Methods); the reads were then categorised according to the number of adenosine residues present (Fig 3a). In mock-infected cells at 6 hpi 12% of the reads mapping to miRNAs contained modifications at the 3’ end, dominated by a single adenosine. Only 0.007% of the modified reads contained 5 or more sequential adenosine nts. In comparison, 46% of the reads mapping to miRNAs in cells infected with VACV were modified at the 3’ end, with 19% of the modifications consisting of polyA “tails” 5 nts or longer in length (Fig 3a). Treatment of cells with AraC did not decrease the extent of miRNA polyadenylation (Fig 3a), consistent with the fact that the enzyme responsible for polyadenylation is the RNA polymerase protein VP55 [11] encoded by the VACV early viral gene E1L [36].

Analysis of the individual miRNAs (Fig 3b) revealed that the number of miRNAs with any type of 3’ modification did not vary greatly in response to either VACV infection or AraC treatment. However the type and extent of modification varied dramatically, with modifications consisting of sequential adenosine residues (a polyA tail) being rare in uninfected but common in VACV infected cells. At 6 hpi 147 miRNAs (28%) in VACV infected cells had reads detected with a polyA tails of at least 5 adenosines and 119 (23%) had longer tails of 10 nts or more. AraC treatment alone caused a small increase in the number of miRNAs detected with a polyA tail of at least 5 adenosines or 10 adenosines however the number of reads mapping to these miRNAs was very low

### Interruption of VACV replication after early viral transcription does not impact tailing but arrests the reduction of modified and unmodified miR-27b-3p

**Fig 4:**
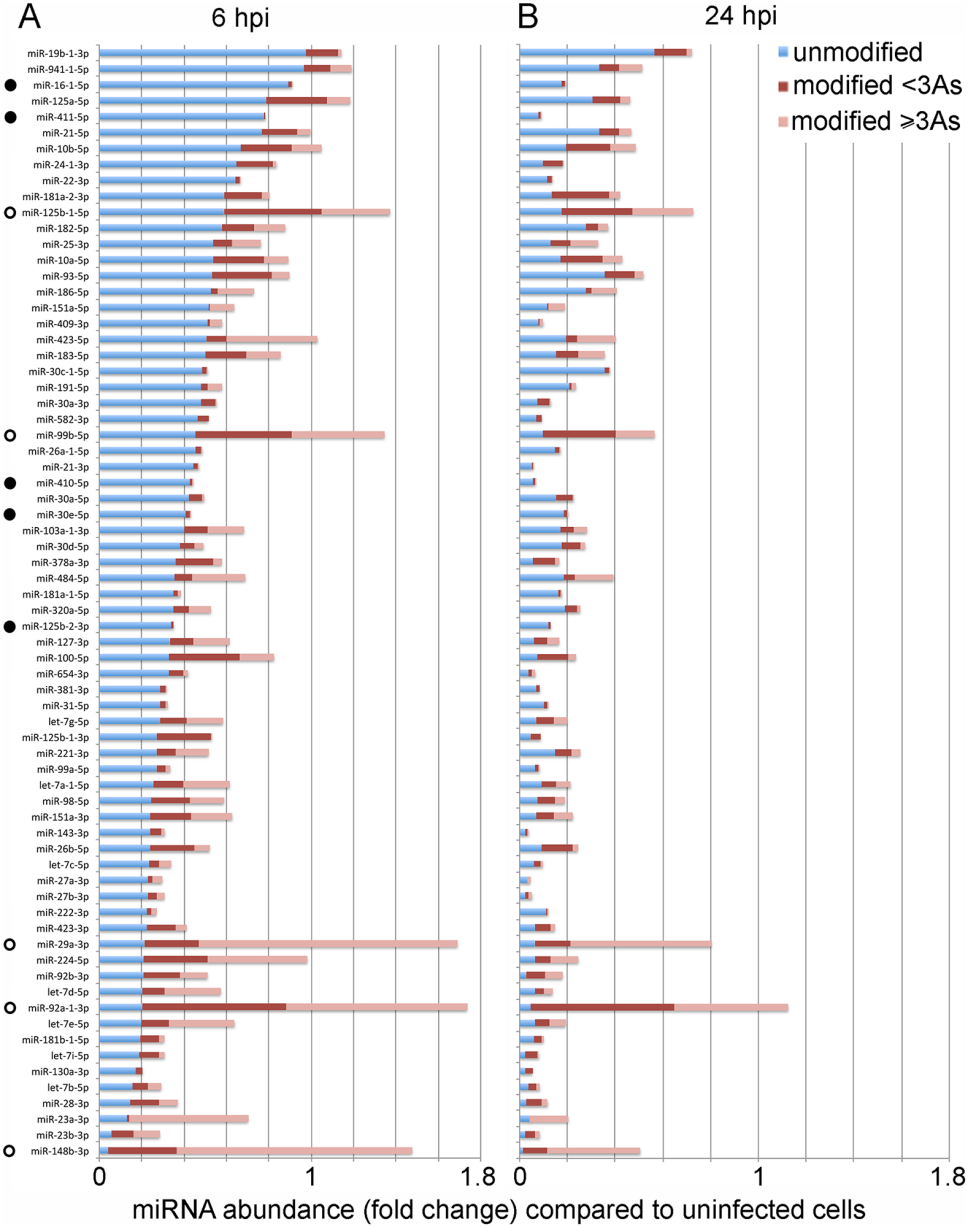
Diversity in the extent of modification and reduction of individual miRNAs upon VACV infection. The proportion of unmodified or modified miRNAs present at (A) 6 hpi and (B) 24 hpi, in relation to uninfected cells. Data for the most abundant 70 miRNAs are displayed, ranked from top to bottom according to largest fold change of the unmodified form at 6 hpi. The top 5 most modified (open circles) or least modified (closed circles) miRNAs at 6 hpi are indicated. The data graphed in this figure are provided in S5 Table.

Quantitative analyses of over a hundred miRNAs in this study reveals striking differences in both the extent of tailing and reduction of individual miRNAs upon infection (S5 Table). When the 70 most abundant miRNAs were ranked according to extent of down-regulation at 6 hpi (Fig 4a, blue bars) the relative proportions of short and long polyA modifications (Fig 4a, red and pink bars) can be seen to vary widely. Some miRNAs showed very high levels of modification (e.g. miR-125b-5p, miR-99b-5p, miR-29a-3p, miR-92a-3p, miR-148b-3p) whilst others showed very low levels of modification (e.g. miR-16-5p, miR-411-5p, miR-410-5p, miR-30e-5p and miR-125b-3p). Furthermore, the extent of modification at 6 hpi (Fig 4a) was not highly predictive of abundance at 24 hpi (Fig 4b). For example, some highly modified miRNAs observed at 6 hpi were still present at high levels at 24 hpi (e.g. miR-125b-5p, miR-99b-5p, miR-29a-3p) and some miRNAs that appeared unmodified at 6 hpi were massively reduced by 24 hpi (e.g. miR-16-5p and miR-411-5p). Thus while there is an overall reduction in mature miRNA levels following VACV infection, the degree of modification and reduction of individual miRNA species shows wide variability, and tailing at 6 hpi is not a clear indicator for reduction by 24 hpi.

**Fig 5:**
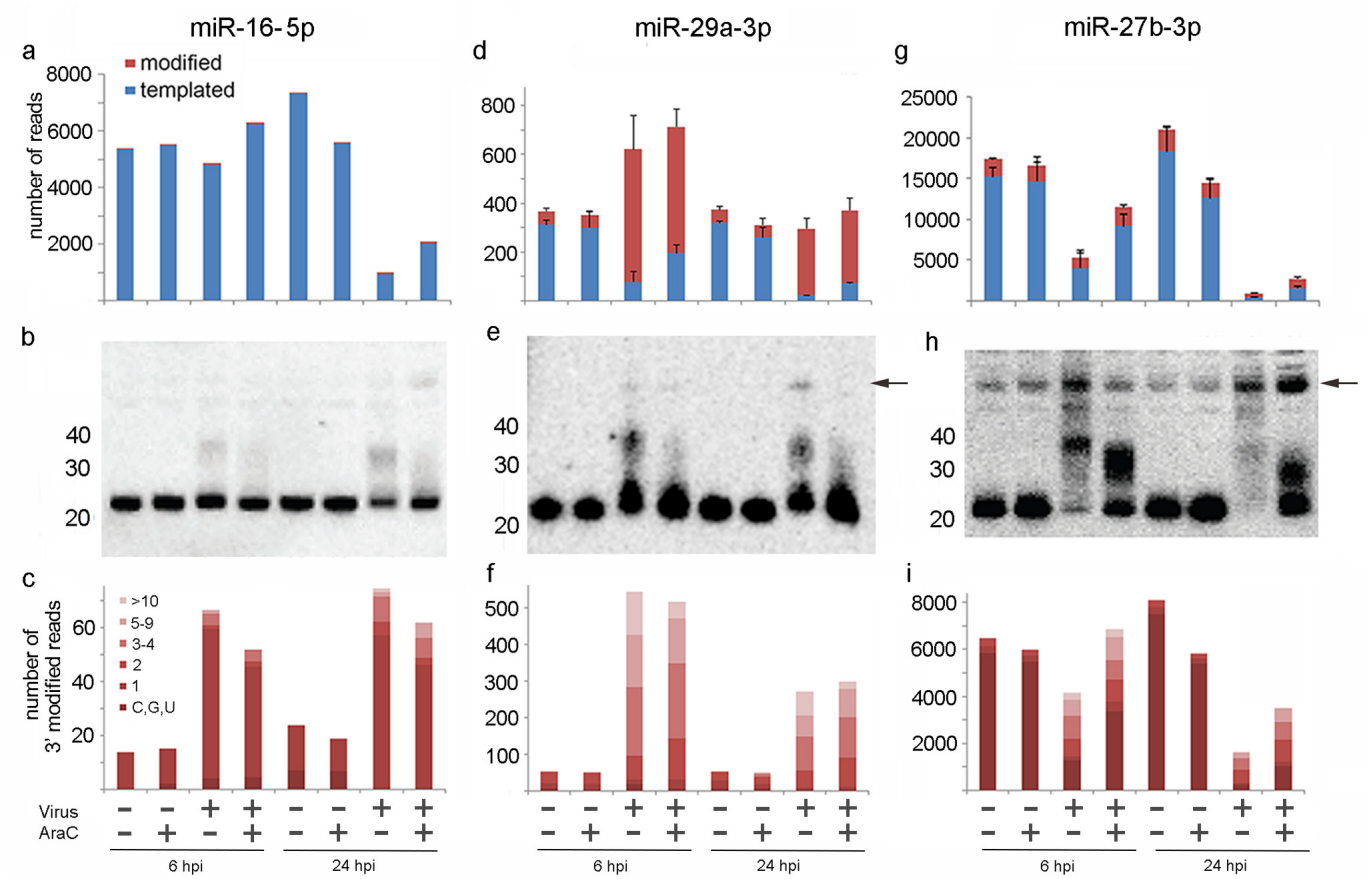
Individual miRNAs show varying susceptibility to VACV-induced polyadenylation and reduction in abundance. The top row shows the relative abundance of unmodified or templated (blue) and modified (red) (a) miR-16-5p, (b) miR-29a-3p, and (c) and miR-27b-3p in uninfected and infected cells at 6 and 24 hpi, with and without AraC treatment. Data shown are the average number of reads for three replicates; error bars represent SD. The middle row shows northern blot analyses of RNA extracted from HeLa cells under the same conditions as in a, d, g, using probes complementary to (b) miR-16-5p, (e) miR-29a-3p, and (h) miR-27b-3p. The arrows in 5e and 5h indicate likely pre-miRNA. The bottom row compares the modifications to the miRNA read for (c) miR-16-5p, (f) miR-29a-3p and (i) miR-27b-3p in uninfected and infected cells at 6 and 24 hpi, with and without AraC treatment.

To visualize the polyadenylation of miRNAs we used northern blotting to examine three highly abundant miRNAs (miR-16-5p, miR-29a-3p and miR-27b-3p) that displayed varying responses to VACV infection. Both sequencing and northern blot showed that the unmodified 22 nt form of miR-16-5p (Fig 5a and 5b) was only modestly down-regulated (10%) in infected compared to uninfected cells at 6 hpi, accompanied by < 1% increase in longer (>/ = 1 nt nucleotide) modified reads (red bars in Fig 5a and 5c) which are visible as a faint lower-mobility smear on the northern blot. After 24 hpi there was a substantial reduction (87%) in abundance of miR-16-5p accompanied by only a 7% increase in modified reads in infected cells (Fig 5c). The reduction in abundance of miR-16-5p at 24 hpi can be partially rescued by AraC treatment. In contrast, miR-29a-3p was extensively polyadenylated in response to VACV infection by 6 hpi but appeared largely resistant to down-regulation (Fig 5d and 5f) with accumulation of numerous polyadenylated species clearly visible in VACV-infected cells. In addition to the modification of mature miR-29a-3p, VACV infection also appeared to result in an increase in the pre-miR-29a-3p (arrow, Fig 5e), suggesting an increase in production or a block in the processing of pri and/or pre-miRNA to mature forms (discussed further below). The third miRNA selected for characterisation by northern blotting was miR-27b-3p (Fig 5g–5i). Although it is likely that the probe used did not distinguish between miR-27a and miR-27b (which differ by only 1 nt) we refer here to miR-27b as this is 16 fold more abundant in Hela cells according to our sequencing analysis (S6 Table) and both family members showed the same pattern of modification and reduction upon infection (S5 Table). VACV infection caused a striking reduction in the amount of unmodified (22 nt) mature miR-27b-3p at 6 hpi, with barely detectable levels at 24 hpi. Polyadenylated miR-27b-3p in virus-infected cells was visible as a dark “smear” on the northern blot with a band at approximately 40 nt (Fig 5h), which would indicate tail lengths of ~ 18 nt. It is important to note that the RNAs were size selected prior to sequencing (Materials & Methods) such that tails longer than this would not be detected, however we see no evidence for their existence by northern blot. Sequence analysis (Fig 5i) detected the majority of miR-27b-3p modifications as 2–9 nt in length, most likely reflecting the difficulty of long homopolymeric sequencing. An increase in abundance of a band consistent with pre-miR-27b-3p was identified in response to VACV infection (arrow), similar to miR-29a-3p. Most strikingly, treatment of VACV-infected cells with AraC had a dramatic effect on both unmodified and modified miR-27b-3p at 6 and 24 hpi, rescuing both forms in infected cells. Thus while miRNA tailing required only early viral gene expression, a full replication cycle was necessary for efficient reduction of unmodified and modified forms of miR-27b-3p.

**Fig 6:**
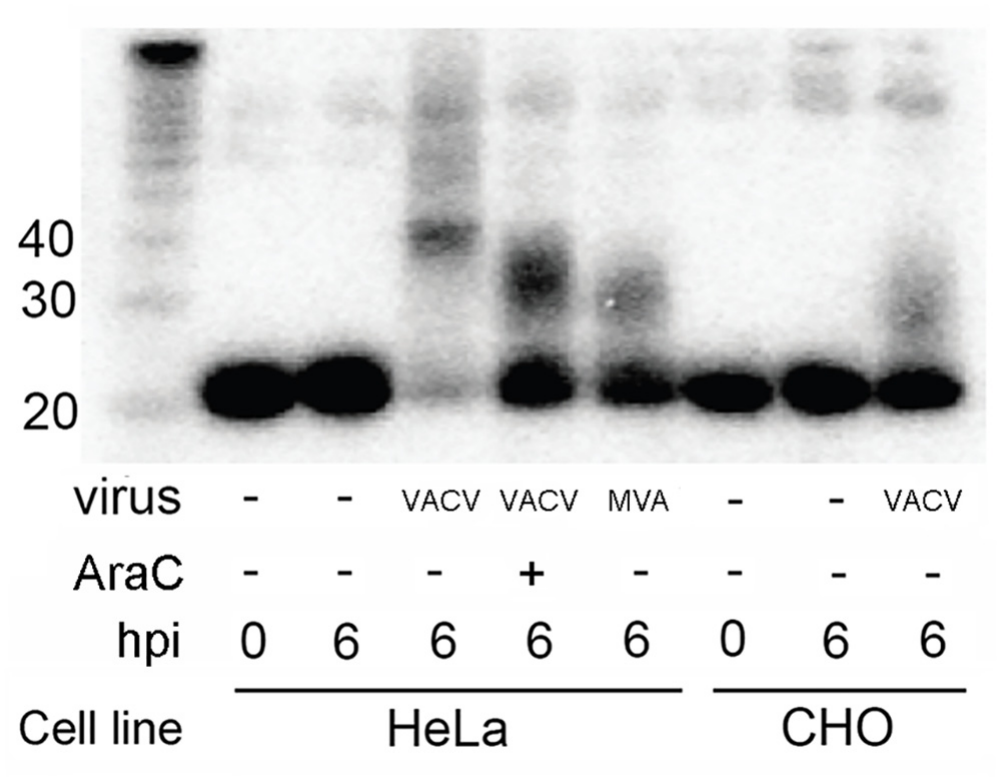
The rapid VACV-induced reduction of modified and unmodified forms of miR-27b-3p is inhibited by arrest of viral replication. Northern blot analysis of RNA extracted from HeLa or CHO cells infected with VACV or MVA at an MOI of 10, or mock infected, in the absence or presence of AraC as indicated. RNA was extracted at 0 or 6 hpi. The probe was a DNA oligonucleotide perfectly complementary to miR-27b-3p.

To examine further the requirement of a full replication cycle on miR-27b-3p reduction, we examined the levels of this miRNA in HeLa cells infected with Modified Vaccinia virus Ankara (MVA), a replication deficient VACV strain lacking 15% of the parental genome. MVA expresses early, intermediate and late genes but is unable to produce infectious progeny virus [37, 38]. We also infected a non-permissive cell line (CHO) with VACV; CHO cells support only early VACV gene expression [39]. In both experiments (Fig 6) polyadenylation occurred but the reduction in abundance of both unmodified and modified species was partially rescued compared to VACV-infected HeLa cells. This suggests that suppression of some host miRNAs by VACV may require the expression of a full complement of intermediate and late VACV genes, many of which are lacking in MVA. Interestingly, in all three conditions where VACV replication is suppressed (HeLa cells infected with VACV and treated with AraC, HeLa cells infected with MVA, and CHO cells infected with VACV) the polyadenylation band has slightly faster mobility compared to HeLa cells infected with VACV, indicating the modified forms are shorter.

### Unmodified miRNAs are shortened in VACV-infected cells

**Fig 7:**
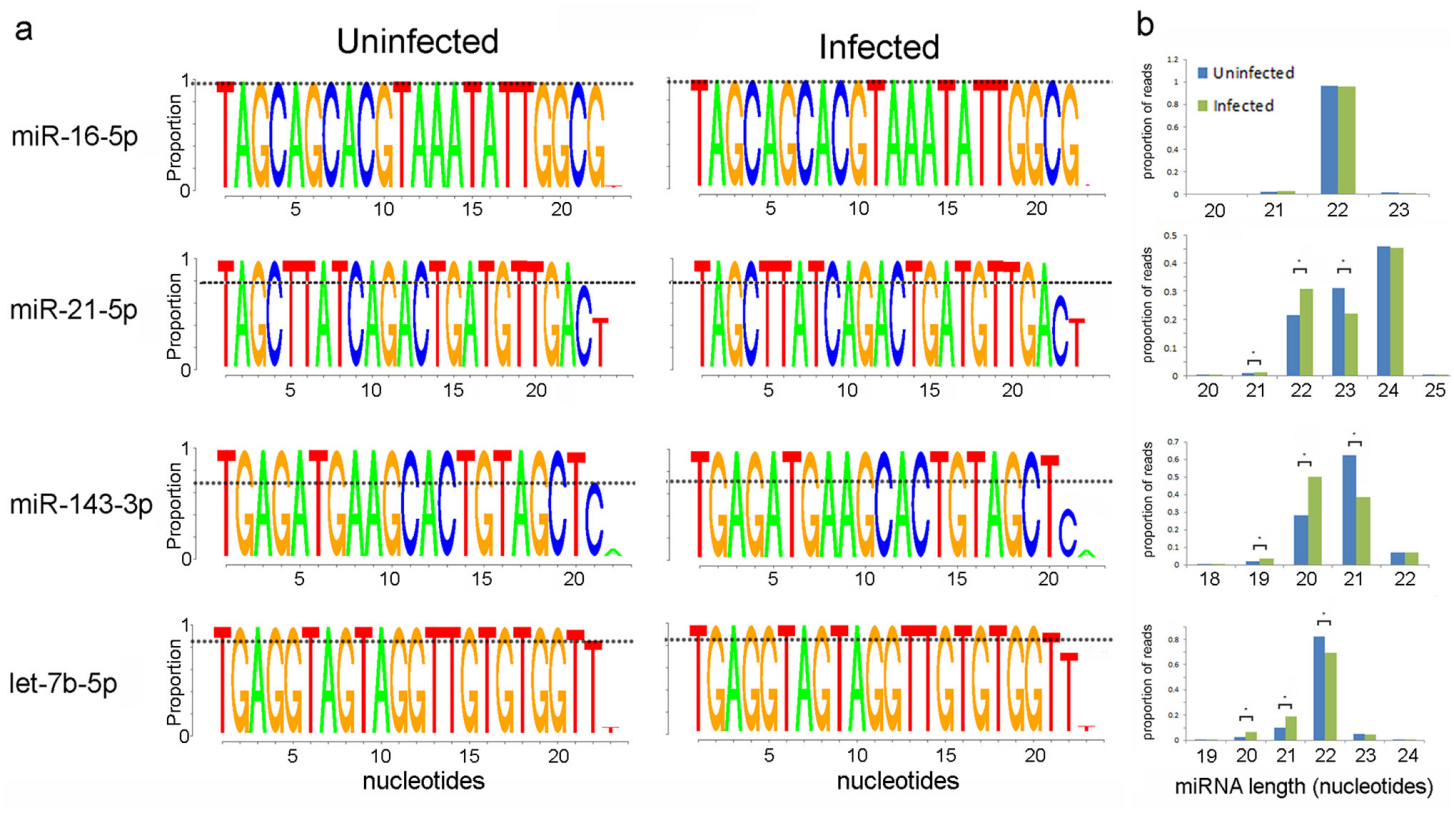
Unmodified miRNAs are shortened in VACV infected cells. (a) Logo images displaying the length of reads mapping to the indicated miRNAs in uninfected cells (left column) and VACV-infected cells (right column). For comparison, a dotted black line has been placed on the images at the same position for uninfected and infected samples in order to visualize the difference between 3’ end composition in uninfected and infected cells, (b) The average proportion (n = 3) of miRNA reads of different lengths in uninfected and infected samples. Statistically significant differences (p<0.05, t-test) are indicated with an asterisk.

Tailing followed by trimming of the 3’ end of miRNAs has been identified as a precursor to miRNA degradation. This has been mechanistically linked to the presence of a highly complementary target that may alter the conformation or accessibility of the 3’ end of the miRNA [23, 24], reviewed in [40]. We do not expect an RNA-mediated recognition mechanism to mediate the widespread degradation in miRNAs observed in VACV-infected cells, however trimming is likely to be a general feature of exonuclease-mediated degradation. It is not possible to infer trimming of polyadenylated miRNAs, however we examined unmodified miRNA sequences for evidence of trimming for ten miRNAs that exhibited either minimal reduction (fold change less than 20%: miR-16-5p and miR-21-5p), intermediate levels of reduction (fold change 40–65%: miR-22-3p and miR-191-5p) or extensive reduction (fold change greater than 70%: let-7b-5p, let-7i-5p, miR-27b-30, miR-29a-3p, miR-31-5p and miR-143-3p) at 6 hpi. The read lengths in uninfected and infected cells were compared and analysed, representative data are shown in Fig 7a and 7b. Overall, a statistically significant increase in the proportion of shorter reads in VACV-infected cells was detected for six of the ten miRNAs, spanning all three classes (miR-21-5p, miR-22-3p, miR-191-5p, let-7b-5p, let-7i-5p and miR-143-3p) (Fig 7 and S4 Fig). Interestingly, the reduction in length was not uniform, for example the proportion of miR-21-5p reads that are 24 nt in length were not affected by VACV infection, however there was a loss in the reads that were 23 nt in length, with concurrent accumulation of the 22 nt forms (Fig 7). This might suggest that only reads of certain lengths are available for tailing, trimming or removal, indicating that the mechanism of miRNA modification employed by VACV is read length dependent. AraC treatment did not affect read length of unmodified miRNAs in either infected or uninfected cells (data not shown). It is worth noting that our analysis will only include reads > 16 nt, therefore extensive trimming would not be detected. In summary, shorter unmodified miRNAs accumulate in response to VACV infection but this feature is not uniform across miRNAs and also not obviously associated with the extent of reduction observed.

## Discussion

Many viruses exert subtle and targeted effects on individual miRNAs[28] however poxviruses appear unique in their ability to directly polyadenylate cellular miRNAs and induce widespread reduction in their abundance[11]. Here we demonstrate substantial variation in the extent of modification and reduction of individual miRNAs induced by VACV, based on a quantitative sequencing analysis of small RNA species at both early (6 hpi) and late (24 hpi) time points. The 6 hour time point is likely to represent a snapshot of the balance between the host anti-viral response and VACV immune evasion mechanisms. At this time the majority of cellular miRNAs are reduced however a subset remain unchanged, including highly abundant miRNAs such as miR-16-5p which modulates the cell cycle [41], the pro-tumorigenic miRNA miR-21-5p [42], and miR-125a-5p which activates the NF-κB pathway [43]. There were even some miRNAs that were significantly up-regulated, including one miRNA, miR-27a-5p, which has been identified as a tumour suppressor in other contexts [44]. The observed diversity in response of the miRNAs to VACV suggests intrinsic differences in the properties of these small RNAs within the cell. These could relate to sub-cellular localization, RISC occupancy, diversity in RISC composition and extent of target binding to individual miRNAs, all of which may be impacted by viral or host factors during infection. These findings also have relevance when considering the potential impact of the cellular miRNAs on different aspects of the VACV life cycle, since some miRNAs remain largely unchanged for at least the first 6h of infection and could regulate genes involved in the viral-host interplay.

The molecular mechanisms underlying the VACV-induced reduction in cellular mature miRNAs are assumed to relate largely to the adenylation of the mature forms, as proposed by Backes et al [11]. Specifically they showed that a synthetic tailed miRNA mimic was absent following transfection into cells whereas a non-tailed version was present, suggesting that the 3’ modification of the synthetic RNA induced its degradation. The exonuclease(s) involved in the degradation mechanism have not been defined and it is not known whether these observations with synthetic RNA would translate to endogenous miRNAs loaded into RISC. We do not observe a clear correlation between the extent of tailing and the extent of reduction of miRNAs. In particular, miRNAs that are highly tailed at 6 hpi are not necessarily among the most reduced at 24 hpi (Fig 4). Since the turn-over rates of miRNAs are known to vary this could contribute to the differential reductions in miRNAs upon infection [18, 24, 45]. Overall our results do not support a model whereby VACV-induced polyadenylation marks all miRNAs for rapid degradation. Differences with the previous report [11] relate mainly to the analysis of more miRNAs in this study and the comparison of both early and late time points. We also demonstrate that some cellular miRNA levels are influenced by intermediate and/or late VACV gene expression. Specifically, AraC treatment partially rescued the levels of modified and unmodified forms of miR-27b-3p (Fig 5). Use of the non-permissive CHO cell line and HeLa cells infected with the mutated VACV strain MVA further supported this result. The difference in miRNA reduction in MVA infected cells compared to wild type was particularly intriguing since MVA does express intermediate and late genes, however it is missing six large sections of genome plus additional smaller mutations when compared to VACV WR. Our results suggest the required factor(s) for miRNA reduction are missing or mutated in MVA. These results also highlight potential pitfalls in extrapolating directly from VACV to MVA with regards to the impact of cellular miRNAs.

Adenosine tails have previously been shown to be added to miRNAs in human cells by the VACV poly(A) polymerase protein VP55, which is expressed early in the viral life cycle [11]. An interesting question is how VP55 selects miRNAs for tailing. Based on sequencing and northern blot analysis here we report a surprising degree of variation in the extent of tailing of individual miRNAs by this protein. The majority of reads of some miRNAs (e.g miR-29a-3p) were tailed whereas others (e.g. miR-16-5p) were largely unmodified. The factors determining the extent of miRNA tailing are unclear but may include subcellular localization, RISC occupancy, turnover rates, accessibility to VP55 and interactions with other effector proteins. Past studies into the specificity of VP55 for viral mRNAs reported that the protein required greater than a 34 nt primer template length before binding to uridylate sequences and catalysing the processive addition of 30 to 35 adenylate residues [46]. Binding of VP55 to various RNAs shorter than 30 nts was not detected [47]. Given that all mature miRNAs are <30 nts, the rules governing VP55 binding to, and polyadenylation of, miRNAs warrant further investigation.

It is unclear whether VACV contributes any factor that could specify the extent to which miRNAs are differentially regulated. It is possible that specificity in this regard simply relates to endogenous miRNA properties in the cell but given that some miRNAs can be advantageous to viruses [48] while others broadly antiviral [49], it is hard to imagine the virus would not evolve to elicit more control over these molecules. At the same time additional reports have demonstrated that VACV also encodes mechanisms for inhibiting pre-miRNA processing [12, 50] which could also contribute to the increase in pre-miRNA forms and reduction of mature miRNAs at late time points that we also observe. In summary this virus interfaces with host small RNA biogenesis and turn over through multiple mechanisms and the functional consequences of this merit further attention.

## Supporting Information

S1 Fig Average number of reads across the eight samples. Each sample is composed of three biological replicates. Error bars represent the standard error of the mean.
(TIF)

S2 Fig A subset of miRNAs increase in response to VACV infection. Comparison of the average abundance of unmodified miRNA (normalised to the total number of high quality reads) in uninfected and VACV-infected cells. N = 3, error bars represent SD, and statistical significance of <0.05 (t-test) is indicated on the figure.
(TIF)

S3 Fig AraC treatment alone does not produce significant changes to miRNA abundance in HeLa cells. Differential expression levels are shown as volcano plots for the 107 most highly expressed miRNAs (>100 reads per million) in HeLa cells with and without treatment with AraC at (a) 6 hpi and (b) 24 hpi.
(TIF)

S4 Fig VACV causes accumulation of shorter read lengths in some miRNAs. The average proportion (n = 3) of miRNA reads of different lengths in uninfected and infected samples are compared. Shorter read lengths of miR-22-3p, let-7i-5p and miR-191-5p accumulate in VACV-infected cells. Statistically significant differences (p<0.05, t-test) are indicated with an asterisk.
(TIF)

S5 Fig Equal loading of samples across gels used for all northern blot analyses was confirmed using ethidium bromide staining. An example of an ethidium bromide stained gel is provided. This gel was used for the northern blots shown in Fig 5e and 5h.
(TIF)

S1 Table Number of small non-coding RNA (sncRNA) reads per sample (three replicates for each treatment). The total sncRNA reads and sncRNA reads matching the human genome are given. Averages and SEM are on the right.
(XLSX)

S2 Table Breakdown of miRNA sequencing reads. (XLSX)

S3 Table Data used to construct volcano figures. (XLSX)

S4 Table Average read number (n = 3) and SD of six miRNAs which increase in amount in response to VACV infection. (XLSX)

S5 Table Abundance and modifications of the 107 most abundant miRNAs at 6 and 24 hpi. (XLSX)

S6 Table Mean, median and standard deviation of unmodified and modified miRNA reads, presented as reads or as abundance (% of total). (XLSX)

## References

[1] Skalsky RL, Cullen BR. Viruses, microRNAs, and host interactions. Annu Rev Microbiol. 2010;64:123–41. Epub 2010/05/19. 10.1146/annurev.micro.112408.134243 20477536 PMC3621958

[2] Bartel DP. MicroRNAs: target recognition and regulatory functions. Cell. 2009;136(2):215–33. Epub 2009/01/27. S0092-8674(09)00008-7 [pii] 10.1016/j.cell.2009.01.002 . 19167326 PMC3794896

[3] Kim VN, Han J, Siomi MC. Biogenesis of small RNAs in animals. Nat Rev Mol Cell Biol. 2009;10(2):126–39. Epub 2009/01/24. 10.1038/nrm2632 . 19165215

[4] Ghildiyal M, Zamore PD. Small silencing RNAs: an expanding universe. Nat Rev Genet. 2009;10(2):94–108. Epub 2009/01/17. 10.1038/nrg2504 19148191 PMC2724769

[5] Krol J, Loedige I, Filipowicz W. The widespread regulation of microRNA biogenesis, function and decay. Nature reviews Genetics. 2010;11(9):597–610. Epub 2010/07/28. 10.1038/nrg2843 . 20661255

[6] Yang JS, Lai EC. Alternative miRNA biogenesis pathways and the interpretation of core miRNA pathway mutants. Mol Cell. 2011;43(6):892–903. Epub 2011/09/20. 10.1016/j.molcel.2011.07.024 21925378 PMC3176435

[7] Grundhoff A, Sullivan CS. Virus-encoded microRNAs. Virology. 2011;411(2):325–43. Epub 2011/02/01. 10.1016/j.virol.2011.01.002 21277611 PMC3052296

[8] Shapiro JS, Varble A, Pham AM, Tenoever BR. Noncanonical cytoplasmic processing of viral microRNAs. RNA. 2010;16(11):2068–74. Epub 2010/09/16. rna.2303610 [pii] 10.1261/rna.2303610 20841420 PMC2957047

[9] Rouha H, Thurner C, Mandl CW. Functional microRNA generated from a cytoplasmic RNA virus. Nucleic Acids Res. 2010;38(22):8328–37. Epub 2010/08/14. 10.1093/nar/gkq681 20705652 PMC3001059

[10] Moss B. Poxviridae: The Viruses and Their Replication In: Knipe DM, Howley PM, editors. Fields Virology. 2. 5 ed: Lippincott Williams & Wilkins; 2007 p. 2906–45.

[11] Backes S, Shapiro JS, Sabin LR, Pham AM, Reyes I, Moss B, et al Degradation of host microRNAs by poxvirus poly(A) polymerase reveals terminal RNA methylation as a protective antiviral mechanism. Cell Host Microbe. 2012;12(2):200–10. Epub 2012/08/21. 10.1016/j.chom.2012.05.019 . 22901540 PMC3782087

[12] Grinberg M, Gilad S, Meiri E, Levy A, Isakov O, Ronen R, et al Vaccinia virus infection suppresses the cell microRNA machinery. Arch Virol. 2012;157(9):1719–27. 10.1007/s00705-012-1366-z . 22674341

[13] Croce CM. Causes and consequences of microRNA dysregulation in cancer. Nature reviews Genetics. 2009;10(10):704–14. Epub 2009/09/19. 10.1038/nrg2634 19763153 PMC3467096

[14] Kim VN, Han J, Siomi MC. Biogenesis of small RNAs in animals. Nat Rev Mol Cell Biol. 2009;10(2):126–39. Epub 2009/01/24. 10.1038/nrm2632 . 19165215

[15] Ruegger S, Grosshans H. MicroRNA turnover: when, how, and why. Trends Biochem Sci. 2012;37(10):436–46. Epub 2012/08/28. 10.1016/j.tibs.2012.07.002 . 22921610

[16] Kai ZS, Pasquinelli AE. MicroRNA assassins: factors that regulate the disappearance of miRNAs. Nature structural & molecular biology. 2010;17(1):5–10. Epub 2010/01/07. 10.1038/nsmb.1762 . 20051982 PMC6417416

[17] Bail S, Swerdel M, Liu H, Jiao X, Goff LA, Hart RP, et al Differential regulation of microRNA stability. RNA. 2010;16(5):1032–9. Epub 2010/03/30. 10.1261/rna.1851510 20348442 PMC2856875

[18] Gantier MP, McCoy CE, Rusinova I, Saulep D, Wang D, Xu D, et al Analysis of microRNA turnover in mammalian cells following Dicer1 ablation. Nucleic Acids Res. 2011;39(13):5692–703. Epub 2011/03/31. 10.1093/nar/gkr148 21447562 PMC3141258

[19] Zhang Z, Zou J, Wang GK, Zhang JT, Huang S, Qin YW, et al Uracils at nucleotide position 9–11 are required for the rapid turnover of miR-29 family. Nucleic Acids Res. 2011;39(10):4387–95. Epub 2011/02/04. 10.1093/nar/gkr020 21288881 PMC3105410

[20] Kai ZS, Pasquinelli AE. MicroRNA assassins: factors that regulate the disappearance of miRNAs. Nat Struct Mol Biol. 2010;17(1):5–10. Epub 2010/01/07. 10.1038/nsmb.1762 . 20051982 PMC6417416

[21] Diederichs S, Haber DA. Dual role for argonautes in microRNA processing and posttranscriptional regulation of microRNA expression. Cell. 2007;131(6):1097–108. Epub 2007/12/18. 10.1016/j.cell.2007.10.032 . 18083100

[22] O'Carroll D, Mecklenbrauker I, Das PP, Santana A, Koenig U, Enright AJ, et al A Slicer-independent role for Argonaute 2 in hematopoiesis and the microRNA pathway. Genes Dev. 2007;21(16):1999–2004. Epub 2007/07/14. 10.1101/gad.1565607 17626790 PMC1948855

[23] Ameres SL, Horwich MD, Hung JH, Xu J, Ghildiyal M, Weng Z, et al Target RNA-directed trimming and tailing of small silencing RNAs. Science. 2010;328(5985):1534–9. Epub 2010/06/19. 10.1126/science.1187058 20558712 PMC2902985

[24] Baccarini A, Chauhan H, Gardner TJ, Jayaprakash AD, Sachidanandam R, Brown BD. Kinetic analysis reveals the fate of a microRNA following target regulation in mammalian cells. Curr Biol. 2011;21(5):369–76. Epub 2011/03/01. 10.1016/j.cub.2011.01.067 21353554 PMC3088433

[25] Ameres SL, Zamore PD. Diversifying microRNA sequence and function. Nat Rev Mol Cell Biol. 2013;14(8):475–88. Epub 2013/06/27. 10.1038/nrm3611 . 23800994

[26] Wyman SK, Knouf EC, Parkin RK, Fritz BR, Lin DW, Dennis LM, et al Post-transcriptional generation of miRNA variants by multiple nucleotidyl transferases contributes to miRNA transcriptome complexity. Genome research. 2011;21(9):1450–61. Epub 2011/08/05. 10.1101/gr.118059.110 21813625 PMC3166830

[27] Landgraf P, Rusu M, Sheridan R, Sewer A, Iovino N, Aravin A, et al A mammalian microRNA expression atlas based on small RNA library sequencing. Cell. 2007;129(7):1401–14. Epub 2007/07/03. 10.1016/j.cell.2007.04.040 17604727 PMC2681231

[28] Libri V, Miesen P, van Rij RP, Buck AH. Regulation of microRNA biogenesis and turnover by animals and their viruses. Cell Mol Life Sci. 2013;70(19):3525–44. Epub 2013/01/29. 10.1007/s00018-012-1257-1 23354060 PMC3771402

[29] Katoh T, Sakaguchi Y, Miyauchi K, Suzuki T, Kashiwabara S, Baba T, et al Selective stabilization of mammalian microRNAs by 3' adenylation mediated by the cytoplasmic poly(A) polymerase GLD-2. Genes Dev. 2009;23(4):433–8. Epub 2009/02/26. 10.1101/gad.1761509 19240131 PMC2648654

[30] Jones MR, Quinton LJ, Blahna MT, Neilson JR, Fu S, Ivanov AR, et al Zcchc11-dependent uridylation of microRNA directs cytokine expression. Nat Cell Biol. 2009;11(9):1157–63. Epub 2009/08/25. 10.1038/ncb1931 19701194 PMC2759306

[31] Friedlander MR, Mackowiak SD, Li N, Chen W, Rajewsky N. miRDeep2 accurately identifies known and hundreds of novel microRNA genes in seven animal clades. Nucleic Acids Res. 2012;40(1):37–52. Epub 2011/09/14. 10.1093/nar/gkr688 21911355 PMC3245920

[32] Libri V, Helwak A, Miesen P, Santhakumar D, Borger JG, Kudla G, et al Murine cytomegalovirus encodes a miR-27 inhibitor disguised as a target. Proc Natl Acad Sci U S A. 2012;109(1):279–84. Epub 2011/12/21. 10.1073/pnas.1114204109 22184245 PMC3252920

[33] Pall GS, Hamilton AJ. Improved northern blot method for enhanced detection of small RNA. Nat Protoc. 2008;3(6):1077–84. Epub 2008/06/10. 10.1038/nprot.2008.67 . 18536652

[34] Yang JS, Phillips MD, Betel D, Mu P, Ventura A, Siepel AC, et al Widespread regulatory activity of vertebrate microRNA* species. RNA. 2011;17(2):312–26. Epub 2010/12/24. 10.1261/rna.2537911 21177881 PMC3022280

[35] Taddie JA, Traktman P. Genetic characterization of the vaccinia virus DNA polymerase: cytosine arabinoside resistance requires a variable lesion conferring phosphonoacetate resistance in conjunction with an invariant mutation localized to the 3'-5' exonuclease domain. J Virol. 1993;67(7):4323–36. Epub 1993/07/01. 8389930 10.1128/jvi.67.7.4323-4336.1993 PMC237803

[36] Jones EV, Puckett C, Moss B. DNA-dependent RNA polymerase subunits encoded within the vaccinia virus genome. J Virol. 1987;61(6):1765–71. Epub 1987/06/01. 3033308 10.1128/jvi.61.6.1765-1771.1987 PMC254178

[37] Meyer H, Sutter G, Mayr A. Mapping of deletions in the genome of the highly attenuated vaccinia virus MVA and their influence on virulence. J Gen Virol. 1991;72 (Pt 5):1031–8. Epub 1991/05/01. . 2033387 10.1099/0022-1317-72-5-1031

[38] Sutter G, Moss B. Nonreplicating vaccinia vector efficiently expresses recombinant genes. Proc Natl Acad Sci U S A. 1992;89(22):10847–51. Epub 1992/11/15. 1438287 10.1073/pnas.89.22.10847 PMC50439

[39] Ramsey-Ewing A, Moss B. Restriction of vaccinia virus replication in CHO cells occurs at the stage of viral intermediate protein synthesis. Virology. 1995;206(2):984–93. Epub 1995/02/01. 10.1006/viro.1995.1021 . 7856109

[40] McCaskill J, Praihirunkit P, Sharp PM, Buck AH. RNA-mediated degradation of microRNAs: A widespread viral strategy? RNA biology. 2015:0 10.1080/15476286.2015.1034912 . 25849078 PMC4615357

[41] Aqeilan RI, Calin GA, Croce CM. miR-15a and miR-16-1 in cancer: discovery, function and future perspectives. Cell death and differentiation. 2010;17(2):215–20. Epub 2009/06/06. 10.1038/cdd.2009.69 . 19498445

[42] Pan X, Wang ZX, Wang R. MicroRNA-21: a novel therapeutic target in human cancer. Cancer biology & therapy. 2010;10(12):1224–32. Epub 2010/12/09. . 21139417 10.4161/cbt.10.12.14252

[43] Kim SW, Ramasamy K, Bouamar H, Lin AP, Jiang D, Aguiar RC. MicroRNAs miR-125a and miR-125b constitutively activate the NF-kappaB pathway by targeting the tumor necrosis factor alpha-induced protein 3 (TNFAIP3, A20). Proc Natl Acad Sci U S A. 2012;109(20):7865–70. Epub 2012/05/03. 10.1073/pnas.1200081109 22550173 PMC3356650

[44] Wu X, Bhayani MK, Dodge CT, Nicoloso MS, Chen Y, Yan X, et al Coordinated targeting of the EGFR signaling axis by microRNA-27a*. Oncotarget. 2013;4(9):1388–98. 23963114 10.18632/oncotarget.1239 PMC3824521

[45] van Rooij E, Sutherland LB, Qi X, Richardson JA, Hill J, Olson EN. Control of stress-dependent cardiac growth and gene expression by a microRNA. Science. 2007;316(5824):575–9. 10.1126/science.1139089 . 17379774

[46] Deng L, Gershon PD. Interplay of two uridylate-specific RNA binding sites in the translocation of poly(A) polymerase from vaccinia virus. EMBO J. 1997;16(5):1103–13. 10.1093/emboj/16.5.1103 9118948 PMC1169709

[47] Gershon PD, Moss B. Uridylate-containing RNA sequences determine specificity for binding and polyadenylation by the catalytic subunit of vaccinia virus poly(A) polymerase. EMBO J. 1993;12(12):4705–14. 7693457 10.1002/j.1460-2075.1993.tb06159.x PMC413915

[48] Jopling CL, Yi M, Lancaster AM, Lemon SM, Sarnow P. Modulation of hepatitis C virus RNA abundance by a liver-specific MicroRNA. Science. 2005;309(5740):1577–81. Epub 2005/09/06. 309/5740/1577 [pii] 10.1126/science.1113329 . 16141076

[49] Santhakumar D, Forster T, Laqtom NN, Fragkoudis R, Dickinson P, Abreu-Goodger C, et al Combined agonist-antagonist genome-wide functional screening identifies broadly active antiviral microRNAs. Proc Natl Acad Sci U S A. 2010;107(31):13830–5. Epub 2010/07/21. 10.1073/pnas.1008861107 20643939 PMC2922253

[50] Chen JS, Li HC, Lin SI, Yang CH, Chien WY, Syu CL, et al Cleavage of Dicer Protein by I7 Protease during Vaccinia Virus Infection. PLoS One. 2015;10(3):e0120390 10.1371/journal.pone.0120390 25815818 PMC4376780

