# Supplementary figures and images for: Quantitative Analysis of MicroRNAs in Vaccinia virus Infection Reveals Diversity in Their Susceptibility to Modification and Suppression
Source: PLoS One. 2015 Jul 10;10(7):e0131787. doi: 10.1371/journal.pone.0131787 (PMC4498801; doi:10.1371/journal.pone.0131787)

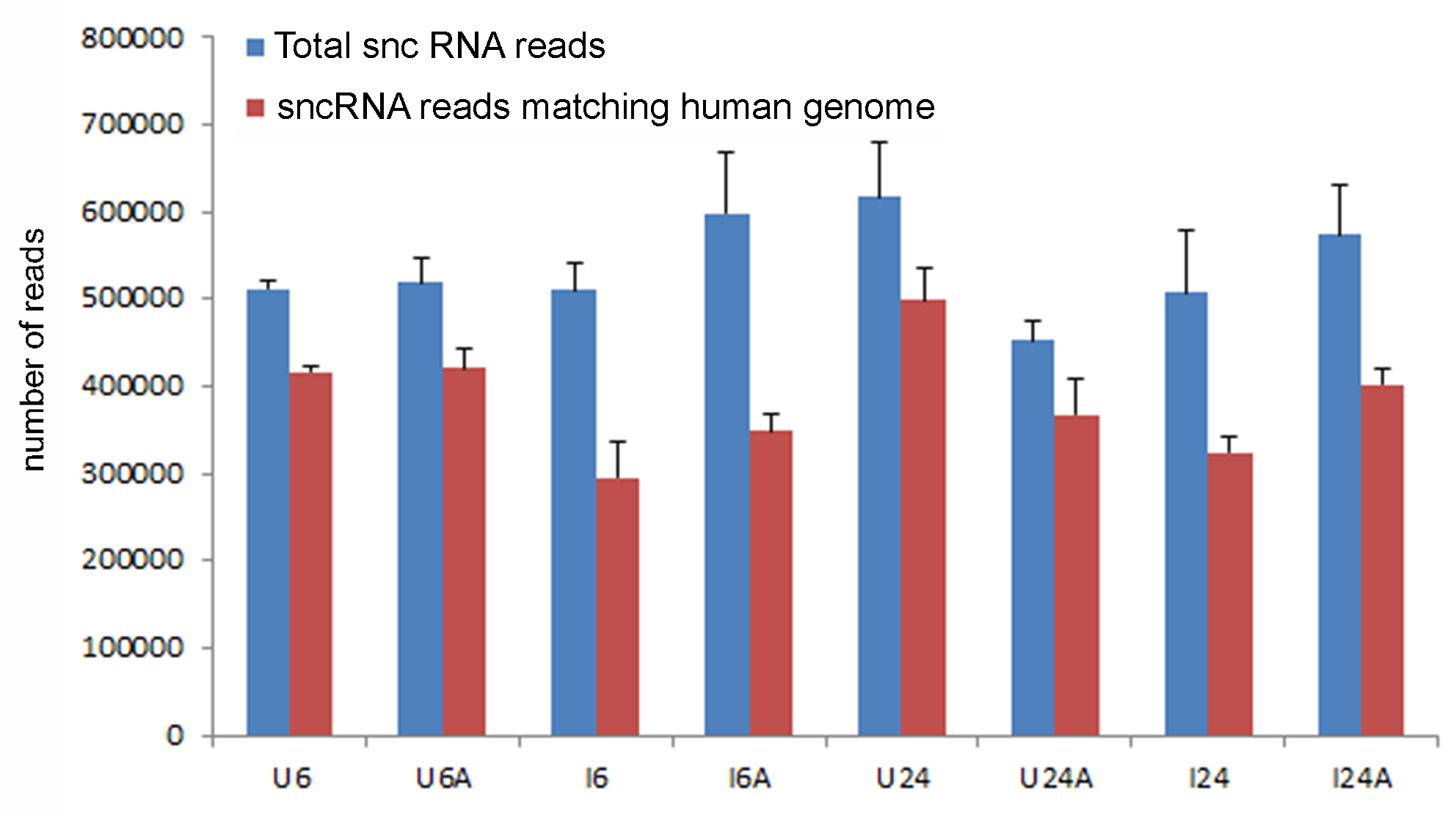

Supplement: S1 Fig — Each sample is composed of three biological replicates. Error bars represent the standard error of the mean. (TIF) [file pone.0131787.s001.tif]

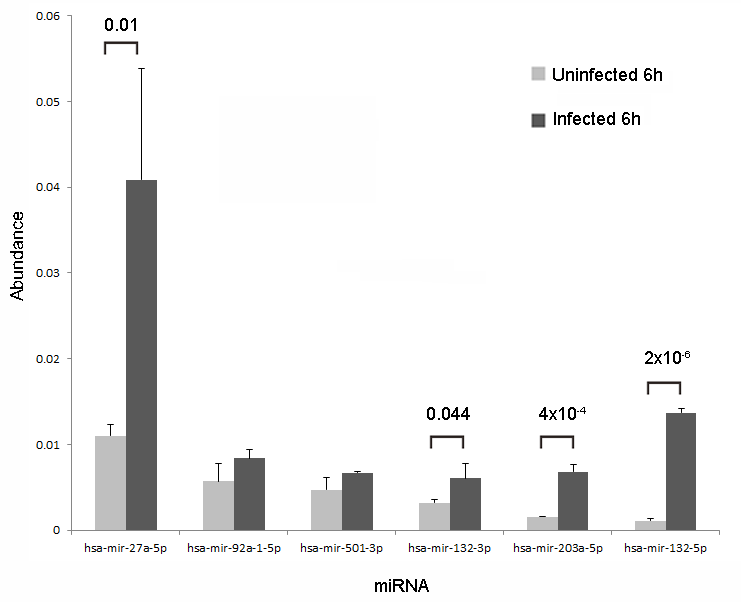

Supplement: S2 Fig — Comparison of the average abundance of unmodified miRNA (normalised to the total number of high quality reads) in uninfected and VACV-infected cells. N = 3, error bars represent SD, and statistical significance of <0.05 (t-test) is indicated on the figure. (TIF) [file pone.0131787.s002.tif]

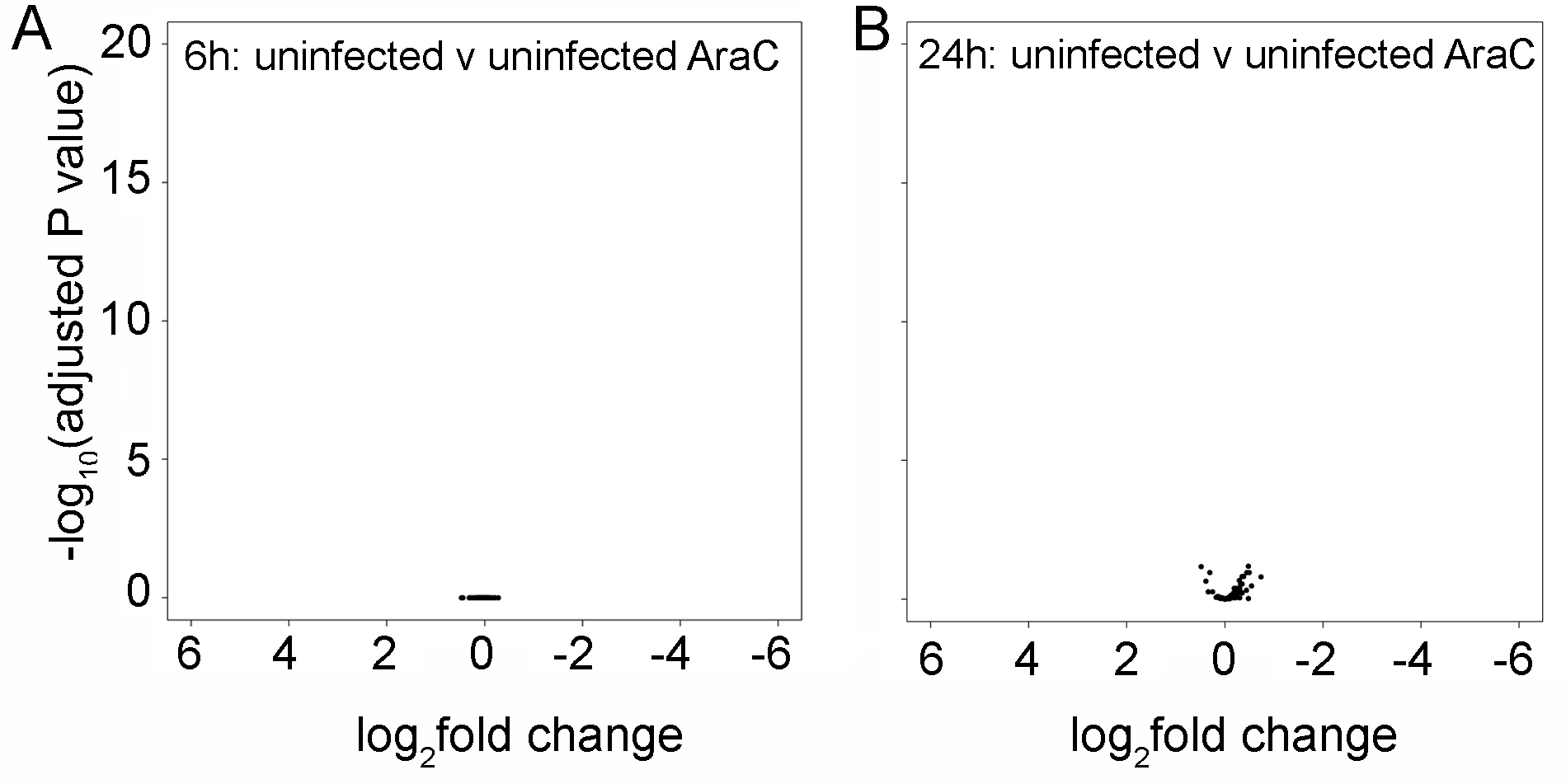

Supplement: S3 Fig — Differential expression levels are shown as volcano plots for the 107 most highly expressed miRNAs (>100 reads per million) in HeLa cells with and without treatment with AraC at (a) 6 hpi and (b) 24 hpi. (TIF) [file pone.0131787.s003.tif]

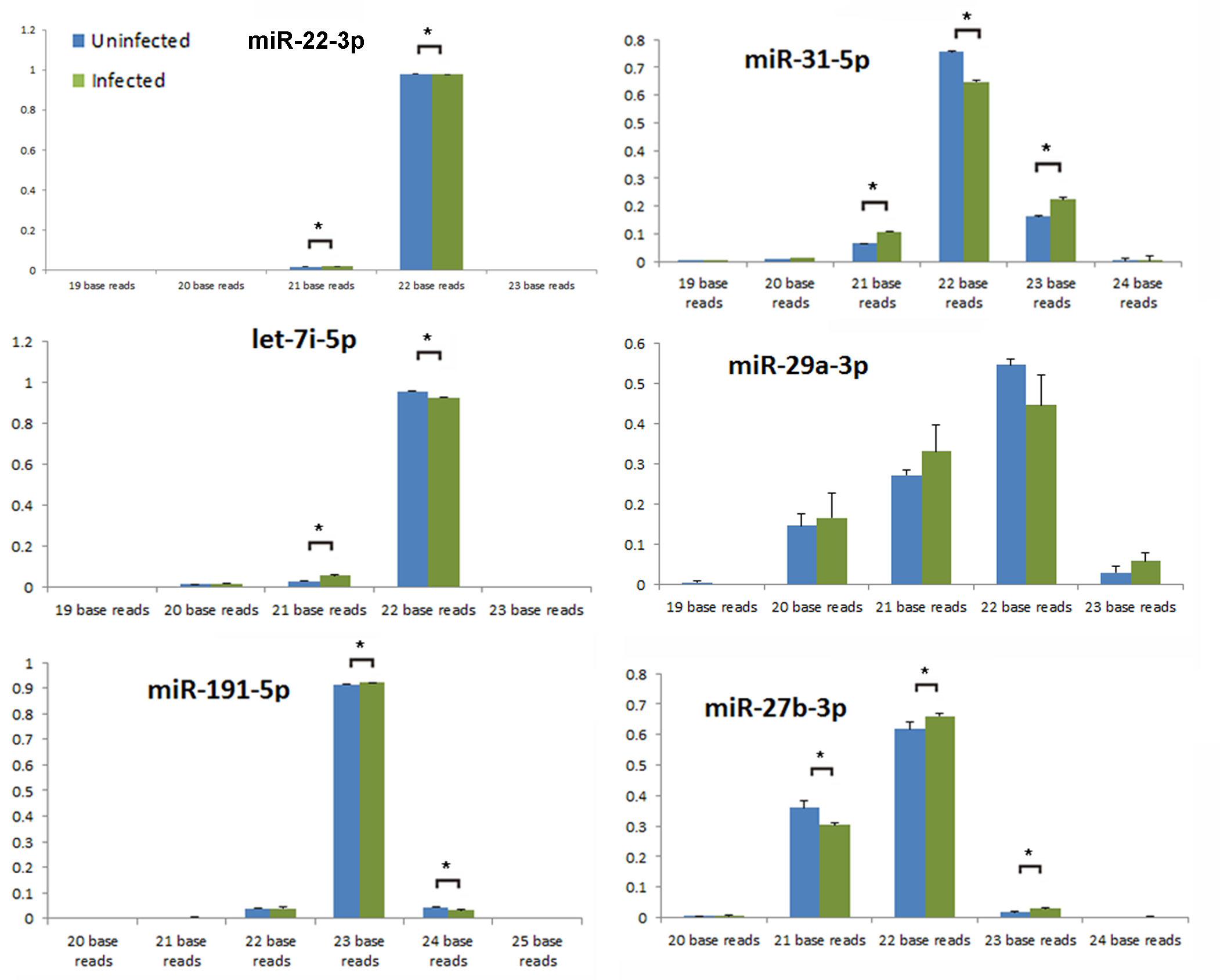

Supplement: S4 Fig — The average proportion (n = 3) of miRNA reads of different lengths in uninfected and infected samples are compared. Shorter read lengths of miR-22-3p, let-7i-5p and miR-191-5p accumulate in VACV-infected cells. Statistically significant differences (p<0.05, t-test) are indicated with an asterisk. (TIF) [file pone.0131787.s004.tif]

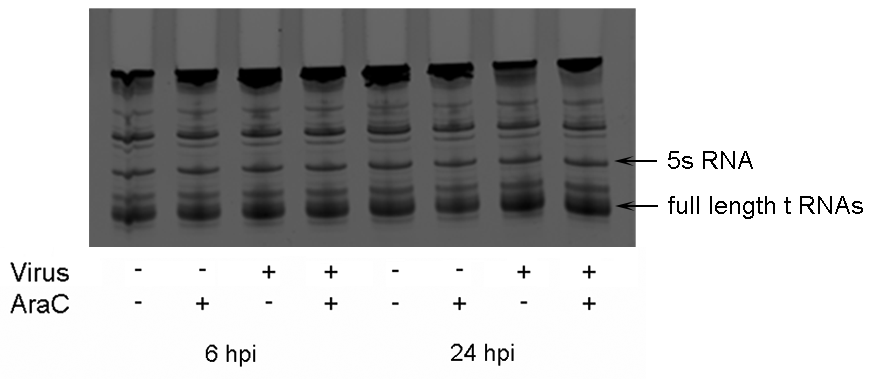

Supplement: S5 Fig — An example of an ethidium bromide stained gel is provided. This gel was used for the northern blots shown in Fig 5e and 5h. (TIF) [file pone.0131787.s005.tif]
